# Ecological risk assessment for land contaminated by petrochemical industry

**DOI:** 10.1371/journal.pone.0204852

**Published:** 2018-10-11

**Authors:** Barbara Gworek, Aneta Helena Baczewska-Dąbrowska, Radosław Kalinowski, Ewa Beata Górska, Hanna Rekosz-Burlaga, Dariusz Gozdowski, Izabella Olejniczak, Magdalena Graniewska, Wojciech Dmuchowski

**Affiliations:** 1 Institute of Environmental Protection–National Research Institute, Krucza, Warsaw, Poland; 2 Warsaw University of Life Sciences—SGGW, Nowoursynowska, Warsaw, Poland; 3 Cardinal Stefan Wyszyński University, Institute of Ecology and Bioethics, Wóycickiego, Warsaw, Poland; Guangdong Technion Israel Institute of Technology, CHINA

## Abstract

Contamination with harmful chemical substances, including organic compounds of the BTEX and PAH groups, constitutes one of the major threats to the functioning of soil habitat. Excessive contents of the above substances can exert adverse effects on soil organisms, reduce biodiversity, and thus deteriorate soil quality. The threat to soil ecosystems within areas particularly exposed to contamination with accumulating chemical compounds was assessed using the Ecological Risk Assessment (ERA) with a multi-stage Triad (triage rapid initial assessment) procedure (taking into account the different lines of evidence). The article presents the results of chemical and ecotoxicological study of soils sampled at sites affected by contamination from petrochemical industry. The study results provided foundations for developing the site specific ERA framework for the area examined.

## Introduction

Contamination of the natural environment due to economic activities leads to an increase in the environmental content of both trace elements and toxic organic compounds [1]. This has particularly serious consequences due to the fact that some of these compounds, e.g. chloroorganic pesticides, polychlorinated biphenyls, dioxins or some polycyclic aromatic hydrocarbons are highly persistent in the environment, and accumulate in soils, bottom sediments and organisms [2–3].

The petrochemical industry uses many chemicals during petroleum processing. Technological processes are the source of not only the intentionally produced compounds, but also of byproducts which are not classified as those produced purposefully. The oil industry was estimated to produce up to 270 billion kilogrammes of hazardous by products per year [4]. Among them, there are numerous polycyclic hydrocarbons, halogenated hydrocarbons, aromatic amines and nitrosamines, as well as organometallic compounds, which have mutagenic potential hazardous to human health [5–6]. C-onsequently, populations living in the neighborhood of petrochemical plants may be exposed to an increased risk of cancer and other adverse health effects [5, 7]. Care has increasingly been taken, over the last years, to reduce negative health effects on people living near industrial complexes. That is why, the research was initiated to determine the levels of pollution of various organic and inorganic compounds as well as the health hazards for people living in the vicinity of petrochemical industry, such as, for example, a large oil refinery located in the south of Europe in the county of Tarragona (Catalonia, Spain) [6].

PAHs are a group of ubiquitous pollutants that are released to the environment owing mainly to anthropogenic factors [8]. Polycyclic aromatic hydrocarbons are by-products of reactions which occur during incomplete combustion of organic matter, and constitute a highly diversified and pervasive group of pollutants, with several hundred chemical compounds [9]. PAHs arise primarily in the course of technological processes of combustion and high-temperature processing of fossil fuels, among other, in power plants, cogeneration power plants, coke industry plants and oil refineries [10].

Moreover, significant amounts of PAHs are released into the environment by transport, as a result of liquid fuel combustion in vehicle and aircraft engines, incineration of municipal and industrial waste, as well as during high-temperature processing of raw materials containing organic matter, such as e.g. oil shale [11]. Some of the polycyclic aromatic hydrocarbons, i.e.: benzo(a)pyrene, benzo(a)anthracene, chrysene, benzo(b)fluoranthene, benzo(k)fluoranthene, indeno(1,2,3,c,d)pyrene and benzo(ghi)perylene exert carcinogenic and mutagenic effect on animal organisms. Considering the varying level of PAHs harmfulness, the PAHs toxicity coefficients (TEF), proposed by the EPA, are typically used to calculate the total toxicity index of PAHs present in soils [12].

Amongst the organic compounds contained in the flue gas, the volatile organic compounds (VOCs) were identified as those having a negative effect on the atmospheric air, including: benzene, toluene, ethylbenzene and xylenes (BTEX) [13]. These compounds are considered as indicators of human exposure to VOCs. Therefore, determining the concentration levels of compounds of the BTEX group is important not only for a general assessment of hazard to human health, but also to the environment [14].

Regulatory and legislative measures have been undertaken, which enable the assessment and/or forecast of environmental changes, and whose aim is to protect the environment, or to take remedial actions to curtail the effects of pollutants' impact on biological resources, ecosystems and human health [15]. The US EPA was the first to publish documents covering the environmental risk assessment procedure [16].

Environmental risk assessment, based on estimating hazards to people and ecosystems, which result from the presence of pollutants in the environment, is a relatively new procedure, now being introduced as a supporting tool for assessing the state of the environment and diagnosing the future environmental hazards [17–18].

The ecological risk assessment procedure allows not only to identify priority areas, but also to determine the order of remediation activities [19]. The assessment is an element of the environmental management decision support system and can be performed together with the health risk assessment or as an independent expert opinion. Assessment of the threat to soil ecosystems using the multi-stage Triad procedure (taking into account the different lines of evidence) seems to be indispensable for the analysis of ecological risk [20–21].

The aim of the work was to develop the ecological risk assessment framework, using the multi-stage Triad procedure (taking into account the different lines of evidence) for the land chemically degraded by the petrochemical industry.

## Study area

The study was made in the area (50°21’01 N, 18°16’35 E) where aromatic hydrocarbons have been produced for almost 80 years. The Authors had permission to conduct the field studies. The owner of the site at the time we collected the samples was Petrochemia—Blachownia S.A. in Kędzierzyn Koźle and all soil samples were collected for its consent. It is the area of the industrial complex of the former "Blachownia" Chemical Plant, where as a result of long-term industrial activity (since the 1930s), the natural soil-forming processes have been degraded. The state of the soil environment was affected by: historical failures and spills, fires (during the Second World War the plant was the target of bombing), incorrect practice of conducting chemical processes in large installations in the years 1950–1970. As a result of the historical activity of chemical plants, the soil environment has been contaminated mainly with aromatic and aliphatic hydrocarbons, phenols and coke benzene refining products. Currently, this plant products mainly: benzene, toluene and solvent naphtha containing xylenes, ethylbenzene and styrene.

The area examined is located in southern Poland on anthropogenic soils in a moderate transitional climate. The area under study is dominated by treeless plant formations in which meadow and grassy vegetation play the dominant role.

**Fig 1:**
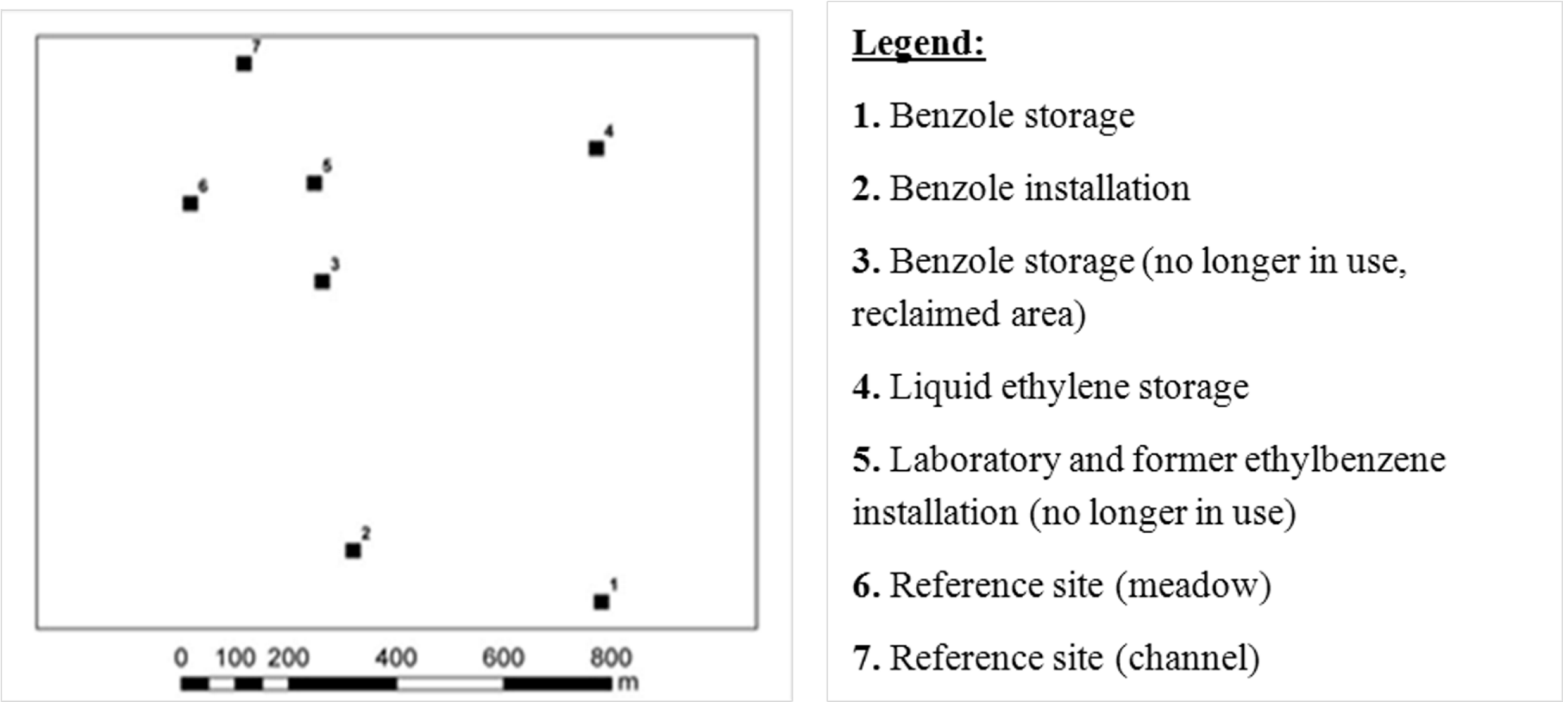
Location of study plots.

Five representative study plots were selected within the area, taking into account the sources of pollution release as well as two reference sites (Fig 1). These two separate control sites were selected in this study, both are in close location (100 and 600 m) to industrial site, but located on the other site of water channel than industrial site, that causes, due to hydrological regime, impossible to transfer pollutions to control site with ground waters. Control sites were also located upwind to the most common wind direction therefore possible transfer of pollutions with the air was limited.

## Materials and methods

### Chemical analyses of soil

The choice of study plots location and, consequently, of soil material sampling sites, was determined considering the variability of soil contamination with organic compounds. The material for chemical analyses was collected from top soil layers (0–20 cm). Soil samples were randomly taken from five points at each of the study plots. To interpret the results, a mixed sample was used, which consisted of all combined samples from a given site. Basic physical, chemical and biological characteristics were determined in the soil material.

In terms of the soil’s physical properties, soil granulometric composition was determined aerometrically as well as dry matter at 105°C. In terms of the basic soil chemical properties, the organic carbon content was determined in the TOC apparatus, pH in H_2_O and KCl–potentiometrically, nitrogen content was determined by Kjeldahl method, hydrolytic acidity by the Kappen method, while the exchangeable cations content, after extraction with 1M ammonium acetate solution at pH 7, by the AAS technique (atomic absorption spectrometry).

Volatile organic compounds, including benzene, ethylbenzene, toluene, meta- and para-xylene, ortho-xylene, styrene, sum of BTEX, sum of xylenes, sum of BTEXS, were determined by a method based on the US EPA 8260, US EPA 5021, US EPA 5021, US EPA 8015 and MADEP 2004 rev. 1.1, ISO 15009 by gas chromatography with the FID and MS detection.

Polycyclic aromatic hydrocarbons (PAHs), including: anthracene, benzo(a)anthracene, benzo(a)pyrene, benzo(b)fluoranthene, benzo(g.h.i.)perylene, benzo(k)fluoranthene, chryzene, dibenzo(a.h)anthracene, indeno(1.2.3.cd)pyrene and naphthalene were determined by the S-PAHGMS01 method based on the US EPA 8270, ISO 18287. Polycyclic aromatic hydrocarbons were extracted with hexane and determined by gas chromatography with the MS or MS / MS detector.

### Phytotoxicity analyses

The Phytotoxkit test of early growth of higher plants was used to assess the phytotoxicity of soil samples, using two dicot plant species: garden cress *Lepidium sativum* and mustard *Sinapis alba* and one monocot species: sorghum *Sorghum saccharatum*. Plant species used in bioassay are standardized monocotyl and dicotyl species frequently used in phytotoxicity analyses of polluted soils and sediments. The assay according to OECD (Organization for Economic Co-operation and Development Organization) was made for samples collected at the study plots and reference sites, as well as for the above plant species. Soil samples were sieved and soaked with distilled water to 100% WHC, placed in a special test plates and covered with blotting paper. 10 seeds of every of the respective species were introduced into each test plate and incubated in the dark for 72 h at 25°C. Following the incubation period, the number of germinated plants was determined, and photographs of the plates were taken. The plant root length was determined using digital image analysis with the application of ImageJ [22]. The inhibition percent was calculated from the formula:

$$I[\%]=\frac{mean\;root\;length\;in\;the\;soil\;OECD-mean\;root\;length\;in\;a\;sample\;}{mean\;root\;length\;in\;a\;sample}*100$$

### Ecological analyses

Soil invertebrates, including springtails are found mainly in the top layer of soil in a layer of roots. Therefore, by the majority of researchers, the samples were taken using steel corer to a depth of 0- 10cm. Springtails *Hexapoda*, *Collembola* were used for ecological analyses. They play an important role in the processes of mineralization and humification of the organic matter. Due to the role of springtails in ecosystems, as well as their response (decrease in abundance, change in dominant structure, species diversity) to pollution, they are considered to be good ecological indicators of health and contamination level of soils. In the laboratory, springtails were extracted from the soil samples in the MacFadyen apparatus, and then preserved in 70% ethylene alcohol. The duration of exposure to light and temperature (25 W bulbs) was 24 h. The springtails were counted and classified into ecological groups based on the characteristics proposed by Christiansen [23], Fjelberg [24–25] and Stach [26]. Ecological risk was analyzed spatially using the Spatial Analysis and Decision Assistance programme, adapted to assess hazards resulting from various factors, not only from radioactive contamination [27].

The programme enables creating a two- and three-dimensional maps of contamination distribution in the screened areas, conducting statistical analyses, developing decontamination scenarios and determining the level of risk for people and the environment [28]. This application is focused on spatial study and designing soil remediation procedures.

Statistical analyses with the aim to determine links between the individual features were performed using correlation analysis and principal component analysis (PCA). The significance level in all analyses was adopted at 0.05. Statistical analyses with the aim to determine links between the individual features were performed using correlation analysis, principal component analysis (PCA) and cluster analysis. The statistical analyses were made with the use of Statistica 13 programme.

## Results and discussion

### Chemical analyses

The physico-chemical characteristics of soils was based on the results of the above described analyses. The soils were segmented into material classes according to the PN-86/B-02480 standard. Depending on the granulometric composition established, there were clay sands in the case of soil samples Nos 2–6, sandy clay in sample No. 1, and loose sand in sample No. 7.

The results of physico-chemical analysis of soils are given in Table 1. The pH values ranged from 4.23 to 7.79. A distinctly higher pH values were found in soils Nos 4 and 5 as compared to the other soils. Large variation in the value of sum of exchangeable base cations (S) implied the variability of the soil sorption capacity (T). The highest value was found in soil No. 4, owing to its alkaline reaction. The soils examined were characterized by a low content of nitrogen (N) and carbon (C). Carbonates (CaCO_3_) were found in two samples solely.

**Table 1:** Chemical properties of test soils.

Sample No	N %	C %	pH H_2_O	pH KCl	S cmol(+)^.^kg^1^	H_h_ cmol(+)^.^kg^1^	T = S+H_h_ cmol(+)^.^kg^1^	V_s_ %	V_h_ ^%^	CaCO_3_%
**1**	0.21±0.04	5.06±0.39	5.71±0.12	4.75±0.93	8.15±4.21	5.96±1.21	14.10±2.35	57.77±4.95	42.23±5.21	0.00±0.00
**2**	0.06±0.01	1.71±0.09	5.57±0.09	4.69±0.87	3.63±1.73	4.87±0.99	8.49±1.75	42.68±3.75	57.32±4.96	0.00±0.00
**3**	0.12±0.02	2.59±0.05	6.35±0.15	5.62±0.34	8.82±2.11	2.91±1.14	11.73±3.79	75.16±8.21	24.84±3.21	0.00±0.00
**4**	0.13±0.02	5.15±0.07	7.23±0.21	6.99±1.01	37.48±11.19	1.20±.64	38.68±4.25	96.89±11.13	3.11±0.17	0.84±0.05
**5**	0.02±0.00	0.54±0.04	7.83±0.19	7.79±1.17	8.38±1.34	0.41±0.09	8.79±2.75	95.35±10.95	4.65±0.21	0.13±0.02
**6**	0.08±0.00	1.78±0.08	5.26±0.13	4.23±0.89	1.94±0.36	5.46±1.12	7.40±1.41	26.15±5.45	73.85±7.12	0.00±0.00
**7**	0.03±0.00	0.78±0.04	5.82±0.11	5.04±1.03	1.27±0.21	1.73±0.99	3.00±0.09	42.35±8.21	57.65±5.13	0.00±0.00

N-nitrogen; C-carbon; pH-reaction; S-sum of exchangeable basic type cations; H_h_-sum of exchangeable acid type cations, T-soil sorption capacity; V_s_-degree of soil saturation with basic type cations; V_h_-degree of soil saturation with exchangeable acid type cations

**Fig 2:**
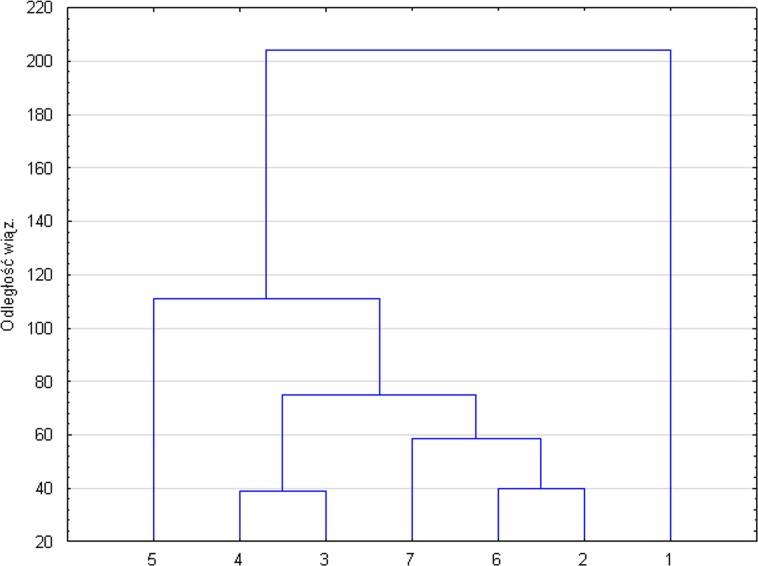
Dendrogram based on cluster analysis, showing similarity of soil samples for all features. Digits 1–7 are assigned to the respective numbers of study plots, from which soil was sampled for analyses.

The similarity of the soils examined with respect to all features is presented in the form of dendrogram (Fig 2). As results from clustering, the highest discrepancies for physical and chemical propertieswere found insoils No. 1 and No. 5 compared to the remaining soils.

The content of volatile organic compounds was found to differ between the study plots examined (Table 2). Considering the individual study plots, volatile organic compounds was detected only in soils found in the vicinity of the active benzole storage site (plot No. 1), and the ethylene plant (plot No. 5). However, no significant effect on the soil VOC content was noted of either the ethylene storage site (plot No. 4) or the benzole installation (plot No. 2).

**Table 2:** Mean content of volatile organic compounds [mg^.^kg^-1^d.m.] in soils from study plots.

	Sample plot No.						
Compound	1	2	3	4	5	6	7
**Benzene**	0.219 ± 0.070	<0.020	<0.020	<0.020	0.710 ± 0.170	<0.020	<0.020
**Ethylbenzene**	<0.020	<0.020	<0.020	<0.020	0.950 ± 0.250	<0.020	<0.020
**Toluene**	0.279 ± 0.070	<0.100	<0.100	<0.100	<0.100	<0.100	<0.100
**Meta & para xylene**	0.165 ± 0.040	<0.020	<0.020	0.035 ± 0.009	<0.020	<0.020	<0.020
**Ortho-xylene**	0.069 ± 0.020	<0.010	<0.010	0.023 ± 0.005	<0.010	<0.010	<0.010
**Sum of BTEX**	**0.732**	**<0.170**	**<0.170**	**<0.170**	**1.660**	**<0.170**	**<0.170**
**Sum of xylenes**	**0.234**	**<0.030**	**<0.030**	**0.058**	**<0.030**	**<0.030**	**<0.030**
**Styrene**	<0.040	<0.040	<0.040	<0.040	<0.040	<0.040	<0.040
**Sum of BTEXS**	**0.732**	**<0.210**	**<0.210**	**<0.210**	**1.660** ±	**<0.210**	**<0.210**

The highest content of volatile organic compounds was determined in the top soil layer sampled from plot No. 1, including: toluene (0.279 mg^.^kg^-1^), meta & para xylene (0.165 mg^.^kg^-1^), ortho-xylene (0.069 mg^.^kg^-1^), BTEX sum (0.732 mg^.^kg^-1^), BTEXS sum (0.732 mg^.^kg^-1^) and sum of xylene (0.234 mg^.^kg^-1^). They were released to the soil from the benzole storage site. On the other hand, no volatile aromatic compounds were detected in the soil sampled from plot No. 3, where the benzole storage used to be a source of contamination in the past, since the area was reclaimed, after the tank had been excluded from use.

The benzene installation (plot No. 2) and the liquid ethylene storage (plot No. 4) did not release VOC into the environment, thus no soil contamination with these compounds was noted. The highest contents of benzene (0.710 mg^.^kg^-1^), ethylbenzene (0.950 mg^.^kg^-1^), BTEX sum (1.660 mg^.^kg^-1^) and BTEXS (1.660 mg^.^kg^-1^) were determined in the soil sampled from plot No. 5, where the former ethylbenzene installation was located. The content of VOC in the other soils fluctuated at the level of values referred to as the so-called geochemical background (Table 3).

**Table 3:** Mean PAHs content [mg^.^kg^-1^d.m.] in soils from study plots.

	Sample plot No.						
Compound	1	2	3	4	5	6	7
**Anthracene**	0.293 ± 0.060	0.035 ± 0.010	0.056 ± 0.010	0.134 ± 0.040	0.014 ± 0.003	0.012 ± 0.004	0.024 ± 0.006
**Benzo(a)anthracene**	1.370 ± 0.170	0.115 ± 0.030	0.404 ± 0.090	0.642 ± 0.150	0.036 ± 0.007	0.080 ± 0.024	0.120 ± 0.027
**Benzo(a)pyrene**	1.590 ± 0.390	0.112 ± 0.030	0.367 ± 0.090	0.649 ± 0.160	0.059 ± 0.019	0.117 ± 0.020	0.157 ± 0.060
**Benzo(b)fluoranthene**	2.780 ± 0.810	0.191 ± 0.060	0.579 ± 0.140	1.180 ± 0.240	0.090 ± 0.002	0.226 ± 0.050	0.298 ± 0.070
**Benzo(g,h,i)perylene**	1.140 ± 0.260	0.090 ± 0.030	0.237 ± 0.070	0.844 ± 0.250	0.091 ± 0.022	0.105 ± 0.030	0.106 ± 0.020
**Benzo(k)fluoranthene**	0.946 ± 0.240	0.069 ± 0.020	0.201 ± 0.070	0.390 ± 0.120	0.029 ± 0.009	0.072 ± 0.020	0.076 ± 0.020
**Chryzene**	1.270 ± 0.390	0.104 ± 0.030	0.331 ± 0.120	0.489 ± 0.100	0.037 ± 0.011	0.094 ± 0.020	0.152 ± 0.060
**Dibenzo(a,h)anthracene**	0.241 ± 0.060	0.019 ± 0.005	0.048 ± 0.010	0.102 ± 0.020	0.012 ± 0.003	0.016 ± 0.005	0.018 ± 0.005
**Indeno(1,2,3,cd)pyrene**	1.390 ± 0.320	0.103 ± 0.030	0.277 ± 0.070	0.677 ± 0.180	0.083 ± 0.002	0.103 ± 0.026	0.126 ± 0.030
**Naphtalene**	0.138 ± 0.030	0.028 ± 0.008	0.058 ± 0.020	0.047 ± 0.010	<0.010	<0.010	0.011 ± 0.003
**Sum of PAHs**	**11.158**	**0.866**	**2.558**	**5.154**	**0.451**	**0.825**	**1.088**

bold values mean the sum of individual relationships, these are not statistically significant values

The PAHs content varied between soils collected from the selected study plots. Quantitatively, it can be represented in the following series: 1> 4> 3> 7> 2> 6> 5, what proves that the content of soil PAHs depended primarily on the sampling site, and thus on the source of pollutant release.

Plot No. 1 (benzole storage) was characterized by a much higher content of the PAHs compared to the other areas, with benzo(b)fluoranthene having the largest share (24.91%) and benzo(k)fluoranthene–the lowest (0.08%). Likewise, a high content of the PAHs was found in the soil sampled from plot No. 4, where the liquid ethylene storage was the source of contamination. A slightly lower content of PAHs was recorded in the soil from plot No. 3, where the benzole storage (currently excluded from use) was located.

Benzo(b)fluoranthene had the largest share among all the PAHs identified in soils on the study plots, while naphthalene the smallest one. In the remaining soils collected from plots Nos. 2, 5, 6 and 7, the contents of the above compounds were relatively low. Based on the results obtained, it can be stated that soil contamination in the vicinity of the laboratory and the former installation of ethylbenzene was lowest compared to the other investigated locations.

**Fig 3:**
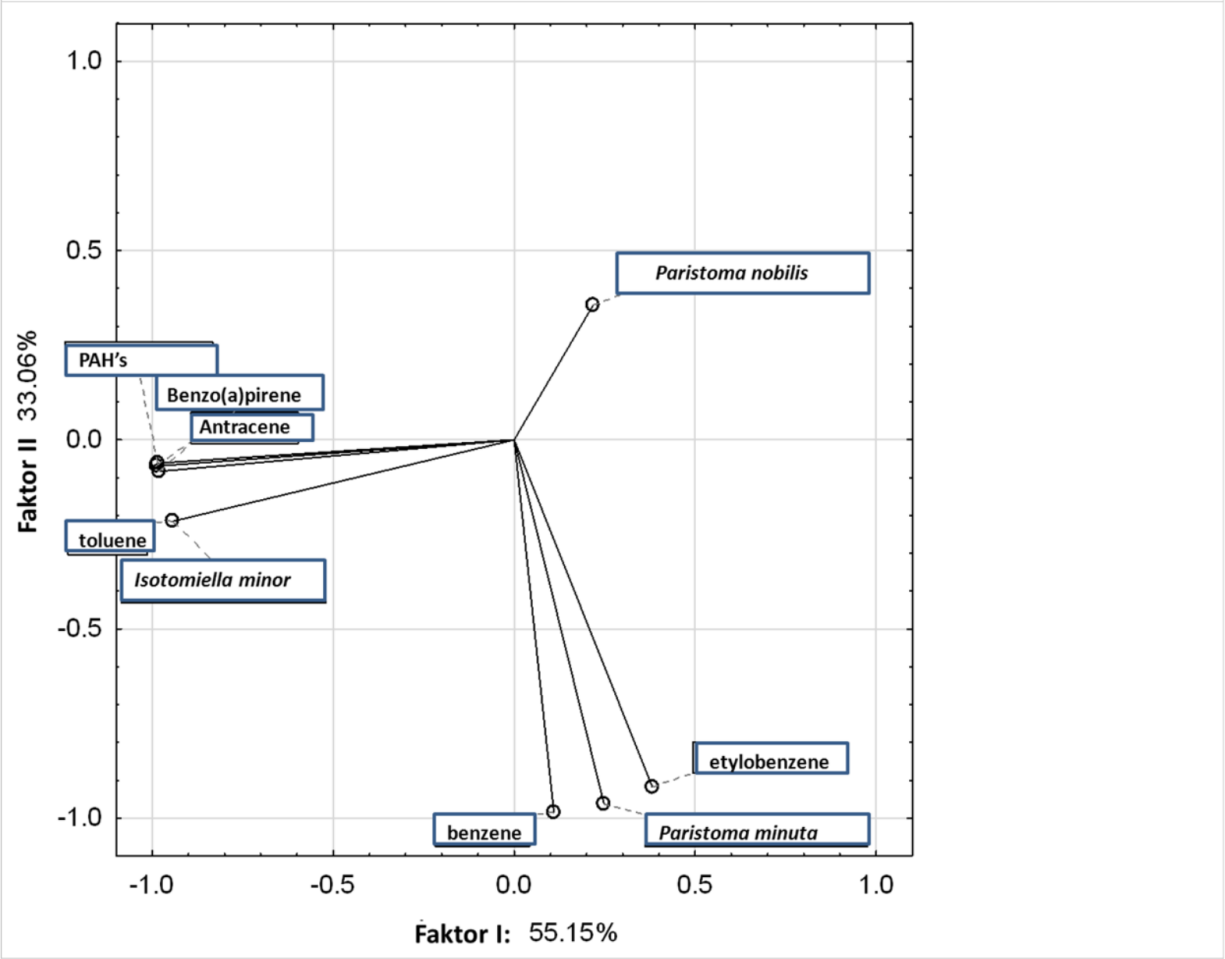
Principal component analysis (PCA) for individual compounds and selected springtail species.

The results of principal component analysis (Fig 3) showed a very strong correlation between anthracene, benzo(a)pyrene and the sum of PAHs (these features overlap in the chart). At the same time, the above three compounds were strongly correlated with toluene, while benzene and ethylbenzene were not related to PAHs.

### Phytotoxicity assessment

Stimulation of root extension was observed in all the plants examined, except for *Lepidium sativum* and *Sorghum saccharatum* growing on soil taken from plot No. 4 whose PAHs content was relatively high (Table 4).

**Table 4:** Inhibition of root growth in plant species: *Lepidium sativum*, *Sinapis alba* and *Sorghum saccharatum* [% effect] grown on test soils.

Plot No	*Lepidium sativum*		*Sinapis alba*		*Sorghum saccharatum*	
	Range	Mean	Range	Mean	Range	Mean
**1**	-29.8 to -0.7	-14.55	-48.0 to-43.5	-45.45	2.8 to -5.6	-1.40
**2**	-7.6 to -26.0	-16.80	-44.5 to -46.3	-45.40	31.4 to 14.2	22.80
**3**	-16.1 to -1.2	-8.65	-57.0 to -26.1	-41.55	-8.2 to -5.4	- 6.80
**4**	11.7 to -2.2.	4.75	-26.4 to 9.2	-8.60	13.7 to 6.0	9.85
**5**	3.7 to -4.0	-0.15	-37.4 to-73.8	-55.60	31.4 to 34.0	32.70
**6**	-5.5 to -3.1	-4.30	-34.0 to-44.2	-39.10	2.0 to 10.9	6.45
**7**	1.2 to -15.9	-7.35	-49.9 to -52.6	-51.25	1.2 to -5.7	-2.25

The root extension stimulation ranged from 0.15% to 55.6% compared to the OECD reference soil. The most likely cause of this phenomenon is a much higher amount of biogenic substances present in the natural soil samples compared to the OECD artificial soil. Mustard turned out to be the most susceptible to soil contamination. In the case of sorghum seedlings, inhibition of root extension for soils from plots Nos 2 and 5 was found to be 22.8% and 32.7%, respectively. Based on the correlation coefficients determined (Table 5), there was a positive relationship between benzene and ethylbenzene in the case of inhibition of root extension in *Lepidium sativum* and *Sorghum saccharatum*. These correlations were negative for the remaining compounds. The inhibition of root elongation decreased with the increase in the content of organic compounds, however, the correlations were insignificant.

**Table 5:** Coefficients of correlation between the content of hydrocarbons examined and the inhibition of plant root extension in *Lepidium sativum*, *Sinapis alba* and *Sorghum saccharatum*.

	Benzene	Ethylbenzene	Toluene	Anthracene	Benzo(a)pyrene	Sum of PAHs
***Lepidium sativum***	0.24	0.38	-0.45	-0.24	-0.25	-0.21
***Sinapis alba***	-0.46	-0.42	-0.14	0.25	0.21	0.26
***Sorghum saccharatum***	0.64	0.73	-0.31	-0.36	-0.43	-0.41

Dicot plants were observed to react differently than the monocot ones, depending on the pollutants tested. Benzene and ethylbenzene stimulated root extension in monocotyls (*S*. *saccharatum*), while anthracene, benzo(a)pyrene and the sum of PAHs–in dicotyls (*S*. *alba*). Both groups of plants reacted similarly only in the case of toluene, however, the correlations were not significant. Similar relations were found by Gworek *et al*. [29], in the study comparing the PAHs uptake by selected species of monocot and dicot plants.

The above authors reported that monocots accumulated PAHs in the roots, to what testifies the significant correlation between the contents of fluorene, phenanthrene and anthracene in the plant material. On the other hand, in dicotyls, where the correlation was negligible, PAHs accumulated both in roots and leaves of plants.

The inhibition of test plants germination was only slightly affected by the diversified content of contaminants in the soil (Table 6). The highest impact on the germination level (17.6% of stimulation) was observed in the case of sorghum in the control soil, sampled from the meadow and from plot No. 3 (reclaimed area). For the remaining soils, a reduction in germination of about 5–10% was determined for the other two species tested.

**Table 6:** Germination of plant species: *Lepidium sativum*, *Sinapis alba* and *Sorghum saccharatum* [% effect] grown on test soils.

Plot No.	*Lepidium sativum*	*Sinapis alba*	*Sorghum saccharatum*
**1**	10	0	5.9
**2**	0	0	-5.9
**3**	5	10	-17.6
**4**	0	10	17.6
**5**	0	10	5.9
**6**	10	0	5.9
**7**	0	0	-17.6

Based on the determined correlation coefficients (Table 7), both positive and negative relationships between individual compounds were found. However, no general trend could be determined.

**Table 7:** Coefficients of correlation between the content of hydrocarbons and germination inhibition in *Lepidium sativum*,*Sinapis alba* and *Sorghum saccharatum*.

	Benzene	Ethylbenzene	Toluene	Anthracene	Benzo(a)pyrene	Sum of PAHs
***Lepidium sativum***	-0.25	-0.32	0.24	0.10	0.17	0.15
***Sinapis alba***	0.36	0.47	-0.35	-0.12	-0.13	-0.10
***Sorghum*** ***saccharatum***	0.48	0.49	-0.04	-0.05	-0.09	-0.07

Phytotoxicity assays are used as environmental tools to assess the contamination of environmental compartments. The impact of soil contamination on plants depends on many factors, such as concentrations and properties of pollutants, exposure time, species of plants and soil properties [30–31]. One of the most important soil properties, which controls the bioavailability of hydrophobic organic pollutants, including PAHs, is the content of organic matter [32].

### Ecological assessment

**Fig 4:**
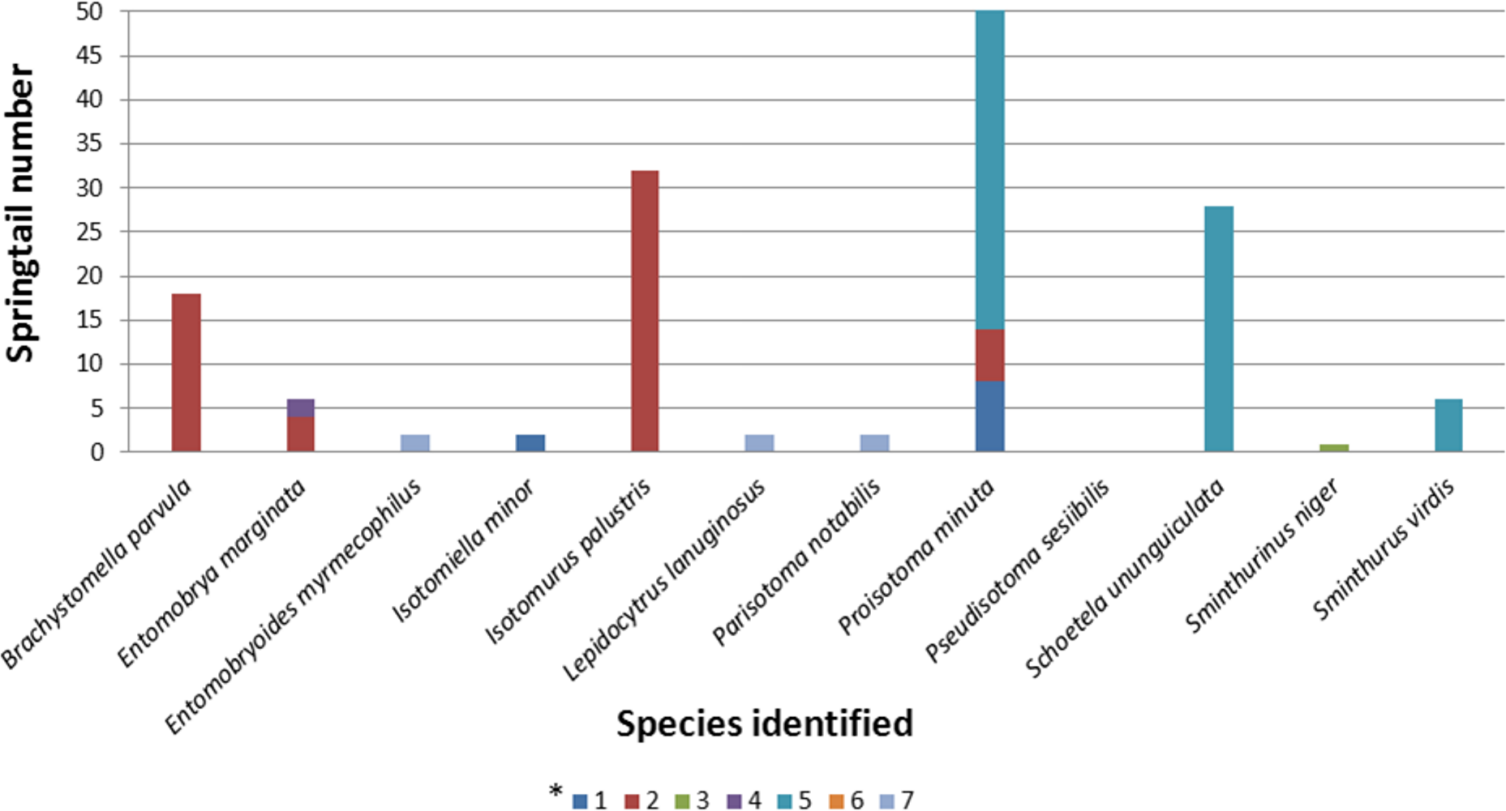
Number of springtail species identified in test soils. Individual colors are assigned to the corresponding numbers of plots where soil was sampled for analysis.

Springtails provide a good tool for assessing hardly measurable and time-varying parameters as well as a source of additional information which facilitates the assessment of ecological effects and trends occurring in the environment [33–35]. The collembols as either single species, various ecological groups or whole assemblages are of indicatory value due to their high sensitivity to changes in soil chemical properties, such as: soil acidification, accumulation of heavy metals and other toxic compounds [36].The occurrence of collembols is also affected by soil pH, humidity, aeration and humus content. Most springtails were detected in soils sampled from plots Nos 2 and 5, while the least in soils from plot No. 3 (Fig 4). Soils sampled from plots No. 1 and No. 7 were found to contain few individuals of *Collembola*. It seems interesting that no springtails were found in one of the reference samples (No. 6), likewise in the reclaimed area (plot No. 3) where they were not detected either.

Based on the correlation coefficients determined (Table 8), it was found that an increase in the content of chemical compounds in the soil resulted in the increase in the numbers of *Isotomiella minor*.

**Table 8:** Coefficients of correlation between the contents of hydrocarbons found in soils and the number of *collembols*.

	Benzene	Ethylbenzene	Toluene	Anthracene	Benzo(a)pyrene	Sum of PAHs
***Brachystomella parvula***	-0.22	-0.17	-0.17	-0.20	-0.26	-0.26
***Entomobrya marginata***	-0.32	-0.24	-0.24	-0.08	-0.17	-0.14
***Entomobryoides myrmecophilus***	-0.22	-0.17	-0.17	-0.25	-0.22	-0.24
***Isotomiella minor***	0.14	-0.17	**1.00**	**0.91**	**0.93**	**0.91**
***Isotomurus palustris***	-0.22	-0.17	-0.17	-0.20	-0.26	-0.26
***Lepidocytrus lanuginosus***	-0.22	-0.17	-0.17	-0.25	-0.22	-0.24
***Parisotoma notabilis***	-0.22	-0.17	-0.17	-0.25	-0.22	-0.24
***Proisotoma minuta***	**0.98**	**0.98**	-0.01	-0.16	-0.18	-0.19
***Schoetella uniunguiculata***	**0.95**	**1.00**	-0.17	-0.29	-0.30	-0.31
***Sminthurinus niger***	-0.22	-0.17	-0.17	-0.11	-0.06	-0.07
***Sminthurus virdis***	**0.95**	**1.00**	-0.17	-0.29	-0.30	-0.31

Values in bold are statistically significant (P<0.05)

There were also significant correlations between the content of benzene and ethylbenzene and the numbers of *Proisotoma minuta*, *Schoetella uniunguiculata* and *Sminthurus viridis*.

However, the interpretation of the results obtained at the present stage is extremely difficult, due to the fact that the springtails reaction to contamination is not unambiguous. For example, Bengtsson *et al*. [37] found that *Collembola* tended to significantly decrease in numbers as a result of soil contamination by heavy metals, whereas the opposite relationship was demonstrated by Migliorini *et al*. [38]. This indicates that the response of collembols to contamination is subject to numerous factors. Environmental conditions can significantly affect the response of springtails, and in particular, soil moisture and temperature, since these invertebrates are particularly sensitive to the above factors [36, 39–40]. The long-lasting contamination may result in an increase in the proportion of parthenogenetic species of springtails having a short life cycle, such as *Isotomiella minor* and *Parisotoma notabilis* [41]. Siepel [42] provided evidence that the above species were abundant in soils contaminated with heavy metals.

The occurrence of PAHs was positively associated with the occurrence of *Isotomiella minor collembols*, while the presence of benzene and ethylbenzene was positively correlated with *Proisotoma minuta collembols*, what may suggest that the above *collembol* species show a low sensitivity to contamination with these compounds (Fig 3). On the other hand, there was a negative relationship between PAHs, benzene and ethylbenzene and the occurrence of *Parisotoma notabilis collembols*.

### Interpretation of results using the SADA programme

One of the most serious threats to the soil habitat functioning, acknowledged by the European Framework Strategy for Soil Protection, is the contamination with harmful chemical substances, including organic compounds from the PAHs group. None of the volatile compounds analyzed, including: benzene, ethylbenzene, toluene, meta- & para-xylene, ortho-xylene, sum of BTEX sum, sum of xylenes, styrene and sum of BTEXS, as well as PAHs (anthracene, benzo(a)anthracene, benzo(a)pyrene), benzo(b)fluoranthene, benzo(g.h.i)perylene, benzo(k)fluoranthene, chrysene, dibenzo(a.h)anthracene, indeno(1.2.3.cd)pyrene and naphthalene) did exceed the permissible national standards. Based on the average content in the sample, various ecological reference scenarios have been traced for individual pollutants, by means of the SADA (Spatial Analysis and Decision Assistance) programme using, among others, the Dutch intervention, EPA R4, EPA RT ESL.

**Fig 5:**
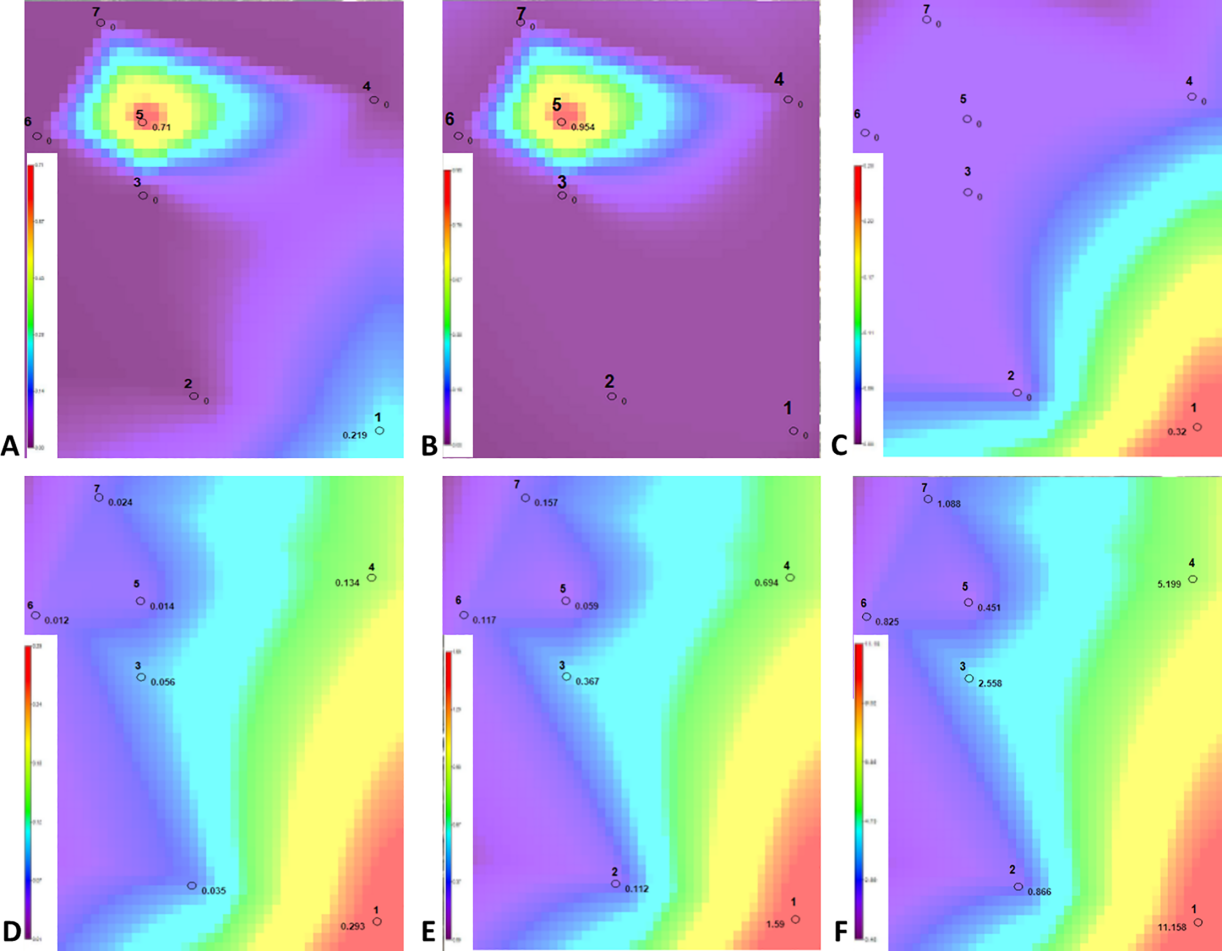
Maps of soil contamination for compounds that exceeded the EPA standards.

It was found that the permissible contents were exceeded for six compounds, including: benzene (A), ethylbenzene (B), toluene (C), anthracene (D), benzo(a)pyrene (E) and the sum of PAHs (F). The results of analyses of spatial contamination of soils are presented in the form of maps with isolines (Fig 5), where individual colours are assigned to specific concentrations of test compounds in the soils. The isolines differentiate the zones of contamination in proportion to the content of individual compounds.

The distribution of isolines in the maps indicates that:

- benzole storage (plot No. 1) is a relatively significant source of local soil contamination with toluene, anthracene, benzo(a)pyrene and total PAHs;
- laboratory and former ethylbenzene installation (plot No. 5) are local sources of relatively heavy soil contamination with benzene and ethylbenzene;
- benzole installation (plot No. 2) and liquid ethylene plant (plot No. 4) have no effect on soil contamination with VOC and PAHs in the area examined, likewise as the site subject to reclamation (plot No. 3) and the reference areas (meadow and channel).

## Conclusion

The range of threats to human health and the soil environment in the examined area was determined by means of the ecological risk analyses using a multi-stage Triad procedure, which combines chemical measurements, ecotoxicological tests and observations of selected soil organisms, together with the use of the SADA programme. However, compared the contents of chemical compounds in the test soils to the values defined by national standards have shown that the area under study should be considered as uncontaminated, and not posing any threat to human health as well as to the basic ecological functions of soils.

The permissible contamination levels were exceeded on some of the soil plots only for three (benzene, ethylbenzene and toluene) from among the nine analyzed volatile organic compounds, and for three (anthracene, benzopyrene and total PAHs) from among the eleven PAHs examined, compared to the EPA and Dutch Intervention standards included in ecological reference scenarios of the SADA programme.

The spatial risk analysis has shown that the sites where sources of pollutant release were located, including: benzole storage (plot No.1), laboratory and the former of ethylbenzene installation (plot No. 5), can be considered as most severely contaminated, while the least contaminated were the sites (meadow and channel) considered as reference (plots Nos 6 and 7).

The pollutants examined have lowered the biodiversity indices and, consequently, deteriorated the soil quality. The Triad procedure, used to assess the ecological risk in the area exposed to PAHs and volatile organic compounds, enabled to identify the zone of a high ecological risk and in need of remediation measures.

Triad approach is a kind of combination of three assessment methods–chemical, ecotoxicological and chemical, each among of them supports another. Because chemical analysis usually does not fully describe site pollution (due to limited number of measured parameters) general–biological related parameters are used. It can be imagined that there are sites with no measured chemical exceedances but with high toxicity toward living organisms and by opposite sites with recognized chemical pollution but non toxic due to i.e. limited bioavailability. In the present paper, it was shown that to assess the threat to soil ecosystems in areas particularly exposed to chemical degradation, the ecological risk assessment methods can be successfully used as a tool for management of lands that threaten human health as well. However, we should remember that remediation of chemically polluted land will be required using of suitable in situ or *ex situ* technologies [43].
